# Selenium Fortification of an Italian Rice Cultivar via Foliar Fertilization with Sodium Selenate and Its Effects on Human Serum Selenium Levels and on Erythrocyte Glutathione Peroxidase Activity

**DOI:** 10.3390/nu6031251

**Published:** 2014-03-24

**Authors:** Attilio Giacosa, Milena Anna Faliva, Simone Perna, Claudio Minoia, Anna Ronchi, Mariangela Rondanelli

**Affiliations:** 1 Department of Gastroenterology, Policlinico di Monza, Monza 20900, Italy; E-Mail: attilio.giacosa@policlinicodimonza.it; 2 Department of Applied Health Sciences, Azienda di Servizi alla Persona (ASP) di Pavia, University of Pavia, Pavia 27100, Italy; E-Mails: milo.milena87@hotmail.it (M.A.F.); simoneperna@hotmail.it (S.P.); 3 Laboratorio di Misure Ambientali e Tossicologiche, Fondazione S. Maugeri, Istituto Scientifico di Pavia, Pavia 27100, Italy; E-Mails: claudio.minoia@fsm.it (C.M.); anna.ronchi@fsm.it (A.R.)

**Keywords:** food fortification, erythrocyte glutathione peroxidise, functional food, rice, trace element, selenium

## Abstract

Selenium food fortification could be a cost-effective strategy to counteract the inadequacy of selenium intake among the Italian population. In this study, the effect of foliar fertilization with sodium selenate of an Italian rice cultivar and the increase of serum selenium and of erythrocyte glutathione peroxidase (GPx) activity after intake of fortified rice, have been evaluated. The effect of foliar fertilization with sodium selenate (50 g Se/ha) *vs.* water was studied. Moreover, in a randomized, double-blind study, 10 healthy women supplemented their usual diet with a daily dose of 80 g of Se-enriched-rice and 10 matched-women with 80 g of regular rice. Before, after 5 and 20 days of supplementation, serum Se and GPx-activity were evaluated. The mean selenium content in Se-enriched-rice was 1.64 ± 0.28 μg/g, while in regular rice it was 0.36 ± 0.15 μg/g (*p* < 0.001). A significant increase of serum Se and GPx-activity was observed only in the intervention group and only after 20 days. The results show that selenium fortification of rice can be achieved with foliar fertilization with sodium selenate and that the 20 days intake of this Se-enriched-rice increases the serum selenium levels and GPx-activity.

## 1. Introduction

Selenium is an essential trace mineral important to human health [1]. Even if the precise biological activity of selenium in humans is still under investigation [2], this mineral constitutes a key component of various selenoproteins involved in enzymatic activities and particularly in redox homeostasis and in thyroid hormone metabolism [3]. A poor selenium status has been associated with increased risk of several chronic diseases, such as cardiovascular diseases [1] and cancer [4]. Prospective studies have generally shown some benefit of higher selenium status on the risk of prostate, lung, colorectal, and bladder cancers, but findings from trials have been mixed, which probably emphasizes the fact that supplementation will confer benefit only if the intake of a nutrient is inadequate [5].

The desirable daily intake of selenium is still debated. The selenium recommended daily allowance (RDA) of the Commission of European Communities is 55 μg/day for the adult European population [6], while the RDA established in the United States is 55 and 70 μg/day for women and men respectively [7]. Multiple studies showed an intake inadequacy of selenium in various Countries: in Europe this appears particularly true for the Italian, Danish and Swedish population [8,9]. Roman Viñas *et al.* analyzed the prevalence of the intake inadequacy of selenium in Europe within the frame of the EURRECA Network of Excellence [8]. This study is based on the analysis of the most representative European data and shows that selenium, folic acid, iodine and vitamin C are the four micronutrients with the highest risk for low diet intake in Europe [8]. As far as selenium is concerned, adults from Finland and from The Netherlands have a prevalence of Selenium inadequacy equal to or below 10% of the population, while it is at or above 30% in the nutritional surveys conducted in Italy, Denmark and Sweden, in adults and in elderly subjects, in males and females [8]. Another study confirms that the average selenium intake in Italian adults hardly reaches the daily recommended level, particularly in the female population [9].

Environmental conditions and agricultural practices greatly affect the selenium content of many foods [10,11,12], thus contributing to the extensive variability that has been observed in selenium intake and selenium status of different populations [13]. In particular, the selenium content in wheat produced in Italy averages on a provincial basis from 7 to 245 ng g^−1^ [14]. The production area of wheat and rice differs considerably in Italy because wheat is produced in most Italian regions, while rice is produced in few provinces mostly located in the north west of the Country and in particular in the counties of Novara, Vercelli and Pavia.

The Italian Institute of Research on Agriculture and Nutrition (INRAN) reports that the rice produced in Italy is characterized by low content of selenium (10 μg/100 g of rice) (INRAN, tables of food composition), mostly due to the low content of selenium in soil of the production area [14]. As a matter of fact, a recent study estimated that the selenium content in the soil of the area where the study has been performed (border between Novara and Vercelli county) is one of the lowest in Italy showing values below 25 μg kg^−1^ [14]. Therefore, on average the selenium content of Italian rice shows values which are too low to meet the demand of this micronutrient. This appears particularly true for people feeding on rice as staple food or for patients with celiac disease or gluten sensitivity who are forced to use rice as a main source of cereals. Moreover, a recent study demonstrated that consumption of white rice was negatively correlated with selenium levels [15]. Therefore, selenium rice fortification could be a possible way to increase selenium intake and to favor the recommended daily allowance (RDA) achievement [6].

Agronomic biofortification represents a food technology which has been used on many occasions with various products, including rice. Rice is one of the most important staple crops and rice fortification with different micronutrients, such as zinc, iron, selenium, has been reported in order to favor human nutrition and health [16,17,18].

The aim of the present study is to evaluate the effect of foliar fertilization with selenate of an Italian rice cultivar in order to produce a selenium fortified rice and to evaluate the increase of serum selenium levels and of erythrocyte glutathione peroxidase (GPx) activity in young healthy subjects supplemented with this selenium enriched rice.

## 2. Experimental Section

### 2.1. Production of Selenium Enriched Rice

The experiment was conducted in the village of Villarboit (Vercelli, Italy), in a well known Italian rice producing area in the north west of the Country, in a region located between Po river and the Alps. The chosen cultivar of rice was “S.Andrea”. Rice sowing was performed in flooded conditions on the 18 April 2012. A first rice fertilization was done before sowing with 60% of nitrogen and potassium needs and a second one was done in the tillering phase, supplying the 20% of needs. A third fertilization was done at the moment of the appearance of the second node in the rice plant, supplying the remaining 20% of nitrogen and potassium. A pesticide treatment was done with Nominee (Bayer CropScience, Milano, Italy) 40 days after sowing. A fungicide treatment was performed with Impact 250 sc (Dow AgroSciences, Bologna, Italy) before the appearance of tassels. Foliar application of sodium selenate at a concentration of 50 g Se/ha was conducted on the 21st of August 2011, when the rice was thoroughly tasseled, seven days after fungicide treatment. No fertilizer or pesticide was supplied after selenium fertilization. Rice harvest was performed on the 29th of September 2011.

Two plots were used for the experiment: each plot was 26 × 26 m and was divided in nine sub-plots of 8 × 8 m with an interrow spacing of 1 m. The intervention (IT) was a unique foliar spraying with 28 mL of sodium selenate (Na_2_SeO_4_) solution at 10% diluted in 30 L of water; while the control treatment (CT) was foliar spraying with 30 L of water.

### 2.2. Rice Selenium Determination

Rice selenium determination was conducted with the method of hydride generation atomic fluorescence spectrometry (AFS). An atomic fluorescence spectrometer (AFS-610, produced by Beijing Rayleigh Analytical Instrument Corp., Beijing, China) was used with a hollow cathode lamp current of 80 mA and a specific procedure has been followed (Environmental Protection Administration, Cincinnati, OH, USA). All measurements were performed in peak height mode. The digested samples were autosampled and determined by AFS. As regards reagents, all solutions were prepared with deionized water. Nitric acid, hydrochloric acid, and perchloric acid of guaranteed reagent grade and potassium hydroxide, potassium tetrahydroborate, and potassium ferricyanide of analytical reagent grade were also used. The standard solution of selenium with a concentration of 1 μg of Se mL^−1^ was prepared with guaranteed reagent grade element selenium dissolved in nitric acid and stored at 0 °C for making the standard curve in the selenium determination. Samples of rice were dried at 50 °C and then milled for selenium determination. Around 1 g of powdered subsample of each sample was taken for analysis. The powdered subsample was put into a high-walled beaker and mineralized with 10 mL of a 4:1 (v/v) mixture of HNO_3_ and HClO_4_ at a constant 150 °C in a sand bath until the solution became colorless and clear. For reduction of Se^6+^ to Se^4+^, 5 mL of 6 mol L^−1^ HCl was added to the digested solution and heated in the same bath at room temperature until the solution became colorless and clear. Then, the digested sample was cooled and diluted to 25 mL with deionized water; 5 mL of the solution was put into a test tube, and 1 mL of concentrated HCl and 0.5 mL of 10% K_3_Fe(CN)6 (w/w) were added; then 1 mL of mixture was autosampled into a reaction vessel. Rice samples and blank were subjected to the same procedure. The data presented here were corrected for blank values, which were usually very low for this method.

### 2.3. Human Study Group

A group of 20 healthy young women (age 25 ± 2 years, Body Mass Index 23 ± 1) was recruited for a 20 day randomized, double-blind intervention study. Ten of them (randomly selected) supplemented their usual diet with a daily dose of 80 g of Se-enriched white S. Andrea rice and 10 were supplied with the same amount of non enriched white S. Andrea rice. The supplementation was done with boiled rice. Before and after 5 and 20 days of supplementation, the fasting serum Se level and the erythrocyte glutathione peroxidase (GPx) activity were evaluated in all the recruited patients. These parameters were also evaluated 10 days after withdrawal of the supplementation. The study design was approved by the Ethics Committee of the University of Pavia and an individual written informed consent was obtained from each subject. Data were gathered from the end of January 2013 to the end of June 2013.

### 2.4. Selenium and GPx Determination in Plasma

Selenium was measured in serum by hydride generating atomic absorption spectrometry (Model 210 VGP, Buck Scientific, East Norwalk, CT, USA) using procedures and methods previously described by other workers [19,20]. The analyst made two measurements for each sample and the average of the observations was recorded as research data. Standard laboratory selenium solutions containing certified selenium content were used as reference materials to control for the quality of the analysis (109915 Titrisol, Merck, Darmstadt, Germany). GPx activity was measured by determining the rate of NADPH oxidation in the presence of 0.25 mmol H_2_O_2_/L [21].

### 2.5. Data Analysis

In order to obtain representative samples of rice, the sampling theory with repositioning was used [22]. Nine lots of enriched and nine of control rice were randomly extracted in a sequential manner [23]. Each lot was derived from one of the nine sub-plots of 8 × 8 m in which the global plot (676 m^2^) has been divided. Each lot of rice underwent an evaluation of the selenium content for three times. The comparison of the amount of selenium in fortified rice and control rice was performed with the Student’s paired *t*-test or Wilcoxon signed-rank test, according to the result of the normality test. Normality was assessed using the Shapiro-Wilk test. For the computation of the sampling plan, the statistical package: PASS 2008-NCSS (Statistical and Power Analysis Software, Kaysville, UT, USA) has been used. For the data analysis, the statistical package: SAS version 9.1 (SAS Institute, Inc., Cary, NC, USA) has been applied.

As far as the statistical analysis of the human data is concerned, results were described as mean and standard deviation if continuous, and as counts and percentage if categorical. Pre-post comparisons were carried out within each treatment group using the paired Student *t* test; mean changes over time and 95% confidence intervals (95% CI) were calculated. Finally, the changes between the treatment groups were compared using a general linear regression model, adjusting for baseline values. Huber White robust standard errors were calculated. The mean difference between the changes and 95% CI was reported. Stata 12 (StataCorp, College Station, TX, USA) was used for these calculations. A 2-sided *p*-value < 0.05 was considered statistically significant.

## 3. Results

The mean selenium amount found in the fortified rice was 1.64 ± 0.28 μg/g of rice, while in the control rice the selenium content was 0.36 ± 0.15 μg/g of rice. The difference between these two data appeared statistically significant (*p* < 0.001).

The serum Se level was significantly increased in the intervention group after 20 days of supplementation with selenium fertilized rice (99.3 ± 17.7 μg/L at time 0 and 114.1 ± 28.6 μg/L at day 20, *p* < 0.003), while it did not change after 5 days (100.4 ± 19.1 μg/L). The control group did not show any significant difference after supplementation with regular rice in both the observation dates (98.2 ± 18.7 μg/L at time 0 and 98.6 ± 18.6 μg/L at day 5 and 100.2 ± 23.4 μg/L at day 20). Similar results were observed when erythrocyte glutathione peroxidase (GPx) activity was evaluated. In effect the group supplemented with Se enriched rice showed a significant increase in GPx activity after 20 days of intervention (19.1 ± 3.1 at time 0 and 22.9 ± 3.0 at day 20, *p* < 0.01), while its value did not change after 5 days (19.3 ± 1.9) and the control group did not show any significant change either after supplementation for 5 or for 20 days (20.2 ± 2.8 at time 0 and 18.4 ± 1.9 at day 5 and 19.1 ± 2.9 at day 20). All these data are reported in Table 1. The follow-up of the supplemented subjects with selenium enriched rice showed that ten days after the end of the intervention, both the mean serum selenium level and GPx activity were reduced as compared to the end of intervention (day 20 of supplementation) and did not differ significantly when compared with the basal values observed before starting the intervention. The serum selenium level and GPx activity observed in the control group 10 days after the end of supplementation with regular rice did not show any significant difference when compared with values observed before and after 5 or 10 days of supplementation, as shown in Table 1.

The inter-group analysis performed after 20 days of rice supplementation showed significant Δ changes of serum selenium levels and GPx activity when intervention and control group were compared, as reported in Table 1. No other inter-group significant differences were observed. The selenium foliar fertilization did not influence the plant growth and yield.

## 4. Discussion

The intake of one portion (80 g) a day of selenium enriched rice for 20 days is associated with a significant increase of serum selenium level and of GPx activity in young, healthy Italian women. This is the main result obtained in this intervention study. A standard serving (80 g) of the fortified uncooked rice used in the present study contains 135 micrograms of selenium, corresponding to 168% of the Italian RDA for adult population. Anyhow, it has to be underlined that, according to our unpublished data, the boiling process of this selenium fortified rice is associated with a 60% selenium loss. Therefore, the final content of selenium in boiled enriched rice is about 0.98 μg/g: this means that 80 g of this boiled rice contain 78.4 μg of selenium. As shown by Roman *et al*., the selenium intake inadequacy (*i.e.*, % of population with values below estimated average required) is 32.8% of the Italian males and 32% of the Italian females [8]. Taking into account these data, and considering that the European Food Safety Authority in 2006 stated that the tolerable upper intake level for selenium is of 300 μg/day, the daily supplementation of selenium due to the intake of 80 g of the enriched rice does not imply any toxicological risk for the Italian population.

Another finding of relevant interest is represented by the correlation between duration of the intervention and effects. As a matter of fact, in this study it has been proven that a significant increase of serum selenium level and of GPx activity is observed after 20 days of supplementation, while a short intervention (5 days) is not adequate to achieve this effect. This information is of great importance from the strategic and nutritional point of view because it offers a useful indication for consumers. Additionally, it has to be considered that the effect reverses quickly: 10 days after the interruption of supplementation with Se enriched rice, selenium serum level, as well as GPx activity, fell to the levels observed before starting the intervention. This finding is in line with previously published data with various selenium enriched food items or supplements [24,25], but no data are available on the serum selenium level and GPx activity after supplementation with selenium enriched rice.

The debate on selenium intake and on serum selenium levels and health is open to discussion [5]. Many epidemiological and laboratory studies show a protective effect of selenium against the development of cancer at numerous sites, including prostate, colon and lung [1,4,5]. Low soil selenium concentration is correlated with cancer mortality throughout the world [26]. Both prospective and case control studies have demonstrated associations between low blood or toenail selenium and increased risk for cancer, particularly of the prostate [27]. The chemopreventive mechanism of selenium remains unclear, but enhanced protection against oxidative stress may be involved [28]. The biological activity of selenium-containing antioxidant enzymes, including glutathione peroxidase and thioredoxin reductase, is influenced by selenium availability [29]. Various preclinical studies have indicated that selenium supplementation can enhance concentrations of the major intracellular antioxidant, glutathione (GSH) and the activity of its rate-limiting biosynthetic enzyme, γ-glutatmyl cysteine ligase, and decrease concentrations of its oxidized products in tissues and blood [30]. GSH is a first line of defense against oxidative stress, and decreased GSH could be associated with increased risk for cancer [31]. Glutathionylated proteins are major products of GSH oxidation, and their formation represents a potential mechanism by which oxidative stress can influence carcinogenesis [32].

As reported by Viñas *et al*., various European Countries, including Italy, show an inadequate selenium intake [8] and a few small-scale surveys of plasma selenium levels evidenced a deficient status in various Italian communities [33]. Foods with the highest Se content are finfish and shellfish products, poultry, beef and pork products, cereal grains and pasta (INRAN). Anyhow, selenium food content is strongly influenced by various factors, primarily by the soil Se content and the Italian soil has been shown to be characterized by low selenium content [14]. As a consequence, the selenium enrichment of a staple food like rice could be useful in increasing the average intake of selenium in Italy, as well as in other Countries with low selenium soil content [34]. This approach has been successfully followed in various European countries, such as in UK and Finland, where the remedy to low dietary Se intake and status has been to enrich locally grown food crops using Se fertilizers (agronomic biofortification) [35].

The selenium enriched rice obtained in this study has been produced with foliar fertilization with sodium selenate of an italian rice cultivar. The methodology used in this experimental model is cheap, easy to realize and allows us to obtain a rice product with a 79% increase of the selenium content as compared to the rice without biofertilization. This finding is in agreement with previous studies [16,17], even though all of them have been conducted in China with Indica cultivars of rice. One of the innovations of this research project is related to the selenium enrichment of a typical Japonica rice cultivar (*S. Andrea cultivar*), which is grown in Italy. The Japonica cultivars are characterized by short and round grains of rice that strongly differ from the Indica rice cultivars commonly grown in Asia, with long and thin grains. Indica and Japonica rice cultivars have similar metabolism; however, the Se enrichment of a Japonica rice cultivar grown exclusively in Europe, and specifically in Italy, has never been done up to now.

## 5. Conclusions

In conclusion, the intake for 20 days of a daily portion (80 g) of selenium enriched rice, obtained by foliar fertilization with sodium selenate, is associated with a significant increase of serum selenium levels and of GPx activity. Therefore, the frequent intake of selenium enriched rice appears potentially useful to favor the achievement of the selenium daily allowances suggested for the Italian population and for other populations with low selenium intake.

**Table 1:** Serum selenium levels and glutathione peroxidase (GPx) activity in basal conditions and after 5 and 20 days of intake of enriched rice or regular rice (placebo) and 10 days after interruption of supply. Values are means ± SD.

Parameters	Supplemented				Placebo			
	Time 0	After 5 Days of Intake of Enriched Rice	After 20 Days of Intake of Enriched Rice	10 Days after Interruption of the Supplementation with Enriched Rice	Time 0	After 5 Days of Intake of Control Rice	After 20 Days of Intake of Control Rice	10 Days after Interruption of Intake of Control Rice
Serum selenium levels (μg/L)	99.3 ± 17.7	100.4 ± 19.1	114.1 ± 28.6 *^,#^	101.2 ± 19.8	98.2 ± 18.7	98.6 ± 18.6	100.2 ± 23.4	97.2 ± 12.7
Serum erythrocyte glutathione peroxidase activity Activity	19.1 ± 3.1	19.3 ± 1.9	22.9 ± 3.0 ^§,#^	19.0 ± 2.3	20.2 ± 2.8	18.4 ± 1.9	19.1 ± 2.9	19.2 ± 1.9

* *p* < 0.03; ^§^ *p* < 0.001: significant comparison between basal and after 20 days of enriched rice ^#^ *p* < 0.01: significant Δ changes when intervention and control group were compared.
